# Mixed Membranes Comprising Carboxymethyl Cellulose (as Capping Agent and Gas Barrier Matrix) and Nanoporous ZIF-L Nanosheets for Gas Separation Applications

**DOI:** 10.3390/polym10121340

**Published:** 2018-12-04

**Authors:** Fang Zhang, Jing Dou, Hui Zhang

**Affiliations:** Jiangsu Provincial Key Lab of Pulp and Paper Science and Technology, Nanjing Forestry University, 210037 Nanjing, Jiangsu Province, China; 13851707389@163.com (F.Z.); dousunnyjing@163.com (D.J.)

**Keywords:** carboxymethyl cellulose, metal–organic framework nanosheets, interactions, gas permeability, gas selectivity

## Abstract

Two-dimensional metal–organic framework (MOF) nanosheets with molecular sieving properties and unique dimensional advantages are highly desired as polymer fillers for gas separation applications. Regarding polymer-supported MOF membranes, it is crucial to enhance the adhesion between the polymeric substrate and the MOF component and avoid MOF particle agglomeration. In this study, hydrophobic, embedded nanoporous nanosheets of a 2D zeolitic imidazolate framework synthesized using zinc salt and 2-methylimidazole (Hmim) aqueous solution (ZIF-L) were incorporated into a carboxymethyl cellulose (CMC) solution to form a steady mixed aqueous suspension through one-step solution blending. This prepared the composite membranes with a fine dispersion of ZIF-L nanosheets (up to loadings of 52.88 vol %) and good adhesion within the highly dense structural CMC matrix due to the strong interactions between ZIF-L and CMC, as confirmed by FTIR, Zeta potential, XPS, and SEM analysis. The potential advantages of CMC over classic polymer matrices used for gas separation mainly include: (a) Good interaction, (b) high dispersion of ZIF-L nanosheets, (c) the gas barrier nature of the CMC membrane, and (d) a facile water-based synthetic process. Based on the molecular sieving effect of ZIF-L and the gas barrier nature of the CMC matrix, gas permeation tests (H_2_, CO_2_, N_2_, CH_4_) of the mixed membrane showed a great improvement in gas selectivities compared with the CMC membrane and the reported pure ZIF membranes.

## 1. Introduction

Advantages, including high mass transfer efficiency as well as easy processing and operation conditions, make membranes competitive gas separation materials in industrial fields [1]. So far, membrane-based H_2_/CO, H_2_/N_2_, and H_2_/CH_4_ separations, air separation, and natural gas purification applications have been used in many industrial fields [2]. Traditional polymer membranes ordinarily have trade-offs between permeable and selective performances [3]. In addition, inorganic porous membranes can be used to separate gas molecules through aperture distinction mechanisms [4]. As a new generation of inorganic molecular sieving materials, metal–organic frameworks (MOFs) are an alternative for gas separation applications due to their well-defined pores and ultrahigh porosity structure. The zeolitic imidazolate framework (ZIF) is a family of MOF that presents zeolite-like structural topologies with excellent thermal and chemical stabilities [5]. ZIF-8 is a stable MOF constructed from tetrahedral zinic ions connected with large cavities of 11.6 Å, which further consist of small apertures of 3.4 Å [6]. ZIF-8 has proved capable of separating smaller gas molecules from larger ones—for instance, separating H_2_ from CH_4_, H_2_ from hydrocarbons, and CO_2_ from CH_4_ [7]. In contrast, a 2D filler with an ultrahigh aspect ratio and anisotropic properties forms a spontaneous “brick-and-mortar” structure inside a polymeric membrane, further effectively interfering with gas diffusion and enhancing the effective diffusivity and selectivity of the mixed membranes [8]. Chen et al. synthesized a 2D zeolitic imidazolate framework (named ZIF-L) using zinc salt and 2-methylimidazole (Hmim) aqueous solution [9]. ZIF-L consists of the same building blocks as ZIF-8, but presents a different structure that stacks into each other along the c axis by H-bonds between 2-methylimidazole molecules. Zhong et al. studied the ZIF-L membrane and its gas permeation properties [10]. Their work examined controlling the orientation of the ZIF-L membrane to achieve better gas separation performance. Liu et al. studied two-dimensional ZIF-L nanosheets and zero-dimensional ZIF-8 nanoparticles incorporated into a sodium alginate (SA) matrix for fabricating mixed membranes used for pervaporation dehydration of ethanol. The mixed membrane presented a brick-and-mortar structure that created water channels and the sieving effect for ethanol dehydration compared to the ZIF-8 crystals’ embedded hybrid membrane [11].

There is increasing interest in the gas separation performance of MOF membranes; however, the problem of gas leakage remains unsolved [12]. This is due to the challenges of preparing an MOF membrane that has defect formation, a lack of interface interactions, or mechanical stability [13]. For a new way to fabricate MOF-based membranes, an MOF/polymer mixed membrane in which MOF is used as the filled component in the polymer substrate has received much attention for gas separation applications. MOF and zeolite membranes possess the same advantages, such as the formation of pinholes/cracks during membrane fabrication. Preparing a no-defect MOF/polymer membrane is a more challenging issue compared to fabricating an MOF membrane [14]. The two major issues in fabricating MOF/polymer mixed membranes include: (1) Poor interactions between MOF particles and the polymer matrix, resulting in low-selective problems; and (2) the aggregation of the MOFs within the polymeric matrix, which decreases the selectivity performance of the membrane [15]. Moreover, almost all MOFs cannot disperse well in water, and MOF/polymer composite membranes have been mainly synthesized using organic solvent. For green synthesis, the choice of a water-based system is preferred.

Cellulose is a perfect natural polymer, as it is a biodegradable, biocompatible, and renewable material [16]. In industrial fields, carboxymethyl cellulose (CMC) is reported to be a viscosity and stability controller [17]. CMC is a polysaccharide that acts on colloidal forces and suppresses the tendency to aggregate solid particles, thus inhibiting flocculation and preventing instability phenomena of particles [18]. The hydrophilic nature of CMC makes its use proper to disperse solid particles in aqueous fluids, while the shear thinning behavior of CMC-based dispersions benefits doctor blade operations [19]. In fact, self-standing and supported membranes consisting of cellulose have attracted interest as gas barrier membranes. In particular, those including surface-charged cellulose fibrils have shown quite low oxygen permeability in dry conditions, superior to those of ethylene-vinyl alcohol copolymers used as commercial oxygen-barrier films [20]. Regarding mixture gas separation, a highly dense structural and surface-charged cellulosic membrane could be used to prevent gas permeation, which makes such a material a potential matrix for an MOF. Matsumoto et al. prepared ZIF-90 cubes embedded in TEMPO-oxidized nanocellulose (TOCN) films and the composite film showed ultraselective CO_2_/CH_4_ separation properties [21]. However, the organic solvents used in ZIF-90 and TOCN are not environmentally friendly. Furthermore, the cubic MOF particles could not provide the high aspect ratio and anisotropic nature for gas molecular diffusion in hybrid membranes.

In this work, a membrane composed of CMC combined with ZIF-L nanosheets was designed and synthesized through a one-step and environmentally friendly water-based process. The results showed that CMC was an effective polymer capping ligand for stabilizing ZIF-L nanosheets in water due to the formation of MOF–CMC complexes in water, thus leading to a composite membrane with a uniform structure. Owing to the molecular sieving property of ZIF-L and the gas barrier nature of the CMC matrix, gas permeation tests of the mixed membrane showed a large improvement in gas selectivities. Moreover, it was proved that CMC is a new class of polymer that can be used as both a capping agent and an adhesive matrix for fabricating MOF-based composite membranes.

## 2. Materials and Methods

### 2.1. Materials

All chemicals were used without further purification. Carboxymethyl cellulose sodium salt (CMC, Sinopharm Chemical Reagent Co., Ltd, Shanghai, China), degree of substitution (DS) 0.84 and medium viscosity (800 mPa·s, 2 wt % in H_2_O), was used as the capping ligand. Zn(NO_3_)_2_·6H_2_O (98%) and 2-methylimidazole (Hmim) (99%) were purchased from Aladdin, Shanghai, China. 

### 2.2. Preparation of CMC Crosslinked ZIF-L Membranes

The preparation of ZIF-L was done according to the procedure reported in [9]. In 200 mL of water, 1.797 g of Zn(NO_3_)_2_·6H_2_O and 5.2 g of 2-methylimidazole (Hmim) were dissolved separately, and then two aqueous solutions were mixed together. The solution was stirred at room temperature for 3 h. The resulting product was washed with deionized water and dried at 80 °C overnight to obtain ZIF-L nanosheets. A certain amount of ZIF-L nanosheets were added into the 2 wt % carboxymethyl cellulose suspension. The mixture turned into a homogenous suspension after ultrasonic treatment in a water bath that was impacted by ultrasonic waves for 20 min. Then, the mixture was allowed to react at room temperature for 3 h. After vacuuming to remove air bubbles, the resulting CMC–ZIF-L solution was cast onto a clean stainless-steel substrate and the mixture was then vacuum-dried at 60 °C to acquire CMC–ZIF-L membranes.

### 2.3. Gas Permeation Tests

The pure gas (H_2_, CO_2_, N_2_, CH_4_) permeability of CMC and CMC–ZIF-L membranes were measured by the variable pressure, constant volume gas permeation cell technique (equipment: Gas Permeate Tester BSG-11A from XiTang Co., Ltd, Guangzhou, China). Each membrane, with a diameter 10 cm, was supported by a filter paper and fixed on the middle intermediate layer. To remove water molecules, the cavity was vacuumed and heated to 50 °C overnight before the test. During the test, the gas feed side was kept at 1 bar and the permeate side under vacuum. The operating temperature was set at 25 °C. The mixture gas permeate performance of the membrane was measured by a calibrated gas chromatograph (GC, Agilent 6890N). During mixed gas permeation, a gas mixture with a ratio of 50/50, 20/80, and 80/20 (vol %) was applied at the feed side of the membrane. In all cases, relative humidity (RH) was carefully controlled during the experiments at 0% RH by dry gas sweeping and vacuuming. Gas permeability values were reported after a steady operating regime was reached.

The ideal selectivity was calculated based on Equation (1):

(1) $$\alpha \left(i/j\right)=p_{i}/p_{j},$$

where *p* is the membrane gas permeability (mL μm m^−2^ day^−1^ kPa^−1^) corresponding to different gas sources. The mixture selectivity was calculated based on Equation (2):

(2) $$\alpha \left(i/j\right)=\frac{y_{i}/y_{j}}{x_{i}/x_{j}},$$

where *x* and *y* are the volumetric fractions of corresponding components in the feed and permeate side. 

### 2.4. Characterization Methods

FTIR spectra were recorded using Bruker Vertex 80 equipment with a detector at 4 cm^−1^ resolution from 500 to 4000 cm^−1^ and 32 scans per sample. The structures of the as-synthesized membranes were characterized by polycrystal X-ray diffraction (PPXRD) using Ultima IV equipment (Xian, China). Powder PPXRD data for crystal structure characterization were recorded in an angular range of 5°–80° at 40 kV and 30 mA. X-ray photoelectron spectroscopy (XPS) measurements were conducted on AXIS UltraDLD equipment (Hongkong, China). Elemental analysis data was determined from the integrated area of Zn 2p, O 1s, and C 1S. Signals with appropriate atomic sensitivity factors were analyzed using XPS software (XPS Peak) for data analysis. Scanning electron microscopy (SEM, JSM-7600F, Beijing, China) was used for morphological characterization. Zeta potential was performed according to the principle of laser doppler electrophoresis on Micrometritics^®^ NanoPlus-2 equipment (Shanghai, China). The mechanical properties of the mixed membranes were characterized via a tensile testing machine (SCT-40) according to the GB/T 1040.3-2006 standard. The thermal stability of the mixed membranes was tested by thermogravimetric analysis (TGA, PerkinElmer STA 6000 thermal analyzer, Shanghai, China). About 10 mg of the sample was used for each testing. The test was done at a heating rate of 10 °C min^−1^ under a nitrogen flow of 20 mL min^−1^.

## 3. Results and Discussion

**Figure 1:**
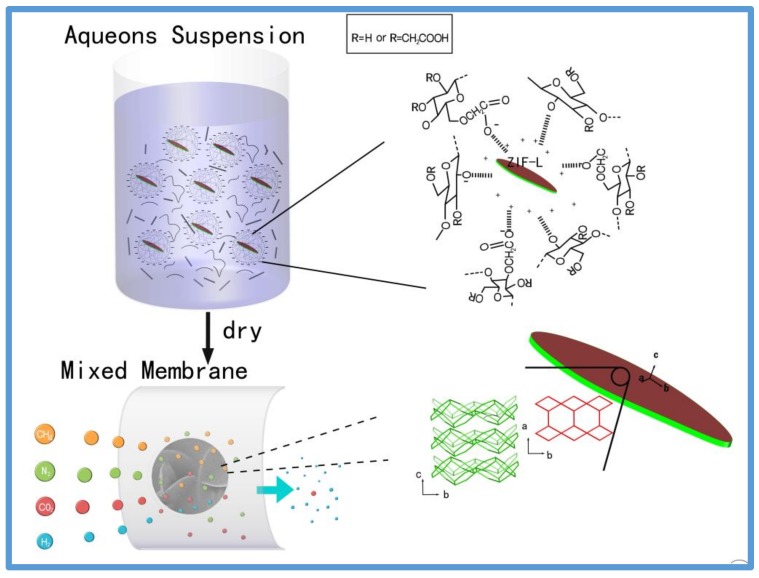
Diagram of CMC-ZIF-L complexes in water and the as-prepared embedded nanoporous composite membrane for gas separation applications.

Based on a CMC crosslinked ZIF-L nanosheets membrane for gas separation, as shown in Figure 1, a facile and organic-solvent-free approach was performed to synthesize uniformly mixed aqueous suspensions, which were then dried to become membrane materials for gas separation. CMC is an anionic, water-soluble polysaccharide derived from cellulose due to their carboxylic groups, as shown in Figure 1. ZIF-L is a 2D ZIF with cavity with dimensions of 9.4, 7.0, and 5.3 Å and a leaf-like shape [10]. ZIF-L possesses the same building blocks as ZIF-8, but has a different morphology. Figure 1 shows the six-membered ring windows and four-membered windows in the [200] plane of ZIF-L. The size of those six-membered ring windows is the same as ZIF-8 and could be estimated to be 3.4 Å from the rigid framework models. The pore size is larger than the kinetic diameter of H_2_ (2.89 Å) and CO_2_ (3.3 Å), but smaller than N_2_ (3.64 Å) [9]. So, ZIF-L membranes possess similar H_2_ permeance and high selectivities of H_2_ over N_2_ and CO_2_ compared with ZIF-8 membranes. In this study, carboxymethyl cellulose was used as a multifunctional polymer ligand to, at the same time, stabilize ZIF-L nanosheets water solutions and prevent particle aggregation. As proposed in this study, CMC generated H-bonds with ZIF-L nanosheets. Moreover, CMC formed Zn–COO coordination links to zinc cations surround the nanosheets, and the formed Zn–COO strongly interacted with the synthesized ZIF-L nanosheets. Thus, adding CMC to a ZIF-L aqueous suspension is the key to ensuring that ZIF-L strongly binds to CMC. Consequently, the dried CMC–ZIF-L membrane showed selective gas permeability for gases with different molecular sizes.

**Figure 2:**
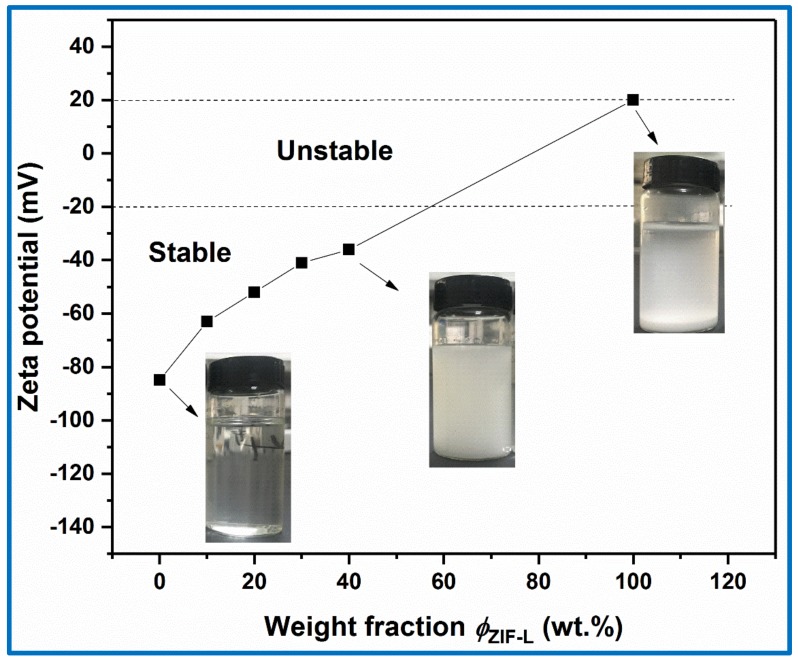
Zeta potential and camera pictures of CMC, and CMC–ZIF-L suspensions.

Zeta potential results also confirmed the interaction between carboxylate groups of CMC and ZIF-L at the interfaces and the effect of these interactions for stabilizing the ZIF-L nanosheets in water. As shown in Figure 2, the CMC solution at a pH of 7.0 presented a zeta potential of –83 mV. The ZIF-L nanocrystals exhibited a positively charged surface with a zeta potential of 20 mV at a pH of 7. After ZIF-L addition, the zeta potentials of the CMC–ZIF-L samples with different component weight ratios were equal to −63, −56, −51, and −47 mV, respectively. According to Riddick (1968), the zeta potential values for a stable solution are higher than 30 or less than −30 mV [22]. It was found that the CMC solution containing anion charges at a pH of 7 were very homogeneous. The decrease of zeta potential of ZIF-L solutions could be attributed to the charge-shielding effect surrounding the MOF nanosheets’ surfaces, resulting in enhanced electrostatic interactions between ZIF-L nanosheets. The loss of negative charges was related to the whole complex effect during the formation of Zn–COO and hydrogen bonds as well as the formation of the CMC–ZIF-L complex. As the reaction proceeded by adding ZIF-L nanosheets, zeta potential values enhanced along with the formation of complex, gradually releasing negatively charged COO^−^ groups in the aqueous solution. The obtained zeta values were lower than the −83 mV value of the CMC solution. However, these values (i.e., <−40 mV) showed that ZIF-L could be electrostatically stabilized by CMC with the negatively charged groups that could be utilized for preventing the agglomeration of the ZIF-L nanosheets.

**Figure 3:**
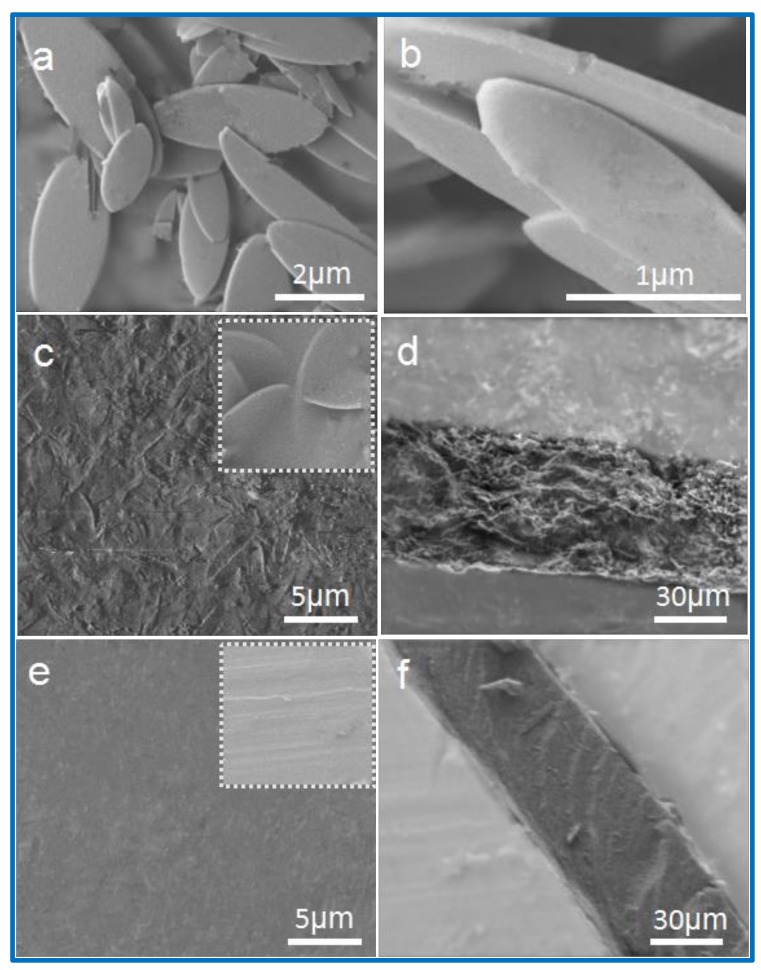
SEM images of (**a**,**b**) ZIF-L nanosheets; (**c**,**d**) CMC–ZIF-L-40 wt % membrane; (**e**,**f**) pure CMC membrane.

Table 1 shows the physical properties of CMC–ZIF-L membranes concerning the component ratio, membrane density, and mechanical strength fabricated from various preparation conditions, which have been presented previously. The densities of the composite membranes (Table 1) were calculated to be between 1.26 cm^−3^ and 1.50 g cm^−3^, which is approximate for the theoretical density (1.28–1.54 g cm^−3^) as calculated according to the loading of ZIF-L. The similar theoretical density and apparent density results indicated that the void volume could be neglected and the conclusion could be verified from SEM images (Figure 3). The volume fraction, *ϕ*_ZL_ (vol %), of ZIF-L in the mixed membrane was calculated according to Equation (3):

(3) $$\phi_{\mathrm{ZL}}\left(\mathrm{vol}\;\%\right)=\frac{m_{D}/\rho_{D}}{m_{D}/\rho_{D}+m_{C}/\rho_{C}},$$

where *m* and *ρ* relate to the mass and density of CMC and ZIF-L, respectively, denoted by subscripts, “*C*” and “*D*” [23]. This further demonstrated that the void volume could be ignored inside the mixed membranes. Hence, the calculated volume fraction was approximate to the actual volume fraction of ZIF-L in the mixed membrane. The membranes were flexible and we found that the mixed membranes with higher contents of ZIF-L nanosheets became white in color and opaque (Figure 7c,d). The mechanical performances of the mixed membranes were evaluated by an electronic stretching device. As illustrated in Table 1, the tensile strength of CMC–ZIF-L mixed membranes decreased along with the ZIF-L loadings up to 40 wt %. Further raising the ZIF-L content caused the decreased tensile strength of the mixed membranes to some extent; the tensile strength of CMC–ZIF-L decreased from 78.1 MPa to 21.5 MPa. The CMC polymer could significantly improve the mechanical strength of the mixed membranes due to the abundant hydrogen bonds among and between the CMC skeleton. Thermal stability is a property of membranes that is highly valued in membrane separation applications. In this work, all the samples could bear the temperature at about 240 °C, as shown in Figure S1, indicating their robustness in temperature-dependent gas separation applications.

The morphologies of the ZIF-L nanosheet crystals and CMC–ZIF-L membrane were evaluated through SEM pictures (Figure 3). SEM images of synthesized ZIF-L nanosheets are shown in Figure 3a,b. The synthesized ZIF-L nanosheets exhibited a unique leaf-shaped morphology with a dimension of 2–5 µm and a thickness of about 100 nm. Nanoporous ZIF-L, with a 2D leaf-shaped morphology, could be beneficial for the fabrication of membranes and composite membranes as gas separation materials. The surfaces of the CMC−ZIF-L membrane are shown in Figure 3c,d; the nanosheets of ZIF-L were individually dispersed on the surface of the mixed membrane. In addition, the ZIF-L nanosheets embedded inside could be observed. The ZIF-L-free CMC membrane showed a tight and smooth texture for the top (Figure 3e) and inside (Figure 3f) surfaces, resulting in the formation of gas barrier layers made from the CMC layer. Carboxymethyl cellulose has a smooth surface, as observed by Wang et al. One of the key issues of an MOF-based composite membrane is to ensure good compatibility between MOFs and polymeric matrices to prevent structure defect issues—for example, small pores and free volume, which are unfavorable for gas separation performance. Compared to traditional polymer matrices, anionic polysaccharide was better able to make them compatible with MOFs. The conclusion could be confirmed by the SEM pictures of the CMC–ZIF-L membranes, which show that no structural defective problems exist, further showing the good compatibility between ZIF-L nanosheets and CMC matrices (Figure 3c,d). Owing to the good dispersion of ZIF-L nanosheets and their interactions with CMC matrices, the obtained CMC–ZIF-L membranes also exhibited good mechanical strength and flexibility, as shown in Table 1 and Figure 7. 

**Figure 4:**
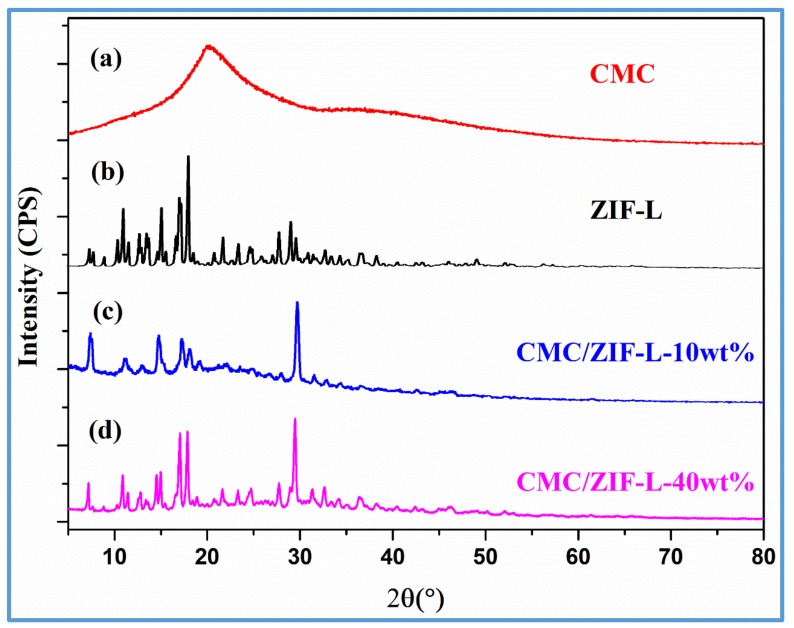
PXRD patterns of the prepared product.

The structures of ZIF-L, CMC, and CMC–ZIF-L hybrid membranes were investigated by PXRD. Figure 4a refers to the PXRD spectrum of the CMC membrane, indicating a unique wide peak of crystalline cellulose at 2*θ* of 22.5°, while the two peaks of pure the CMC membrane at 2*θ* of 14.5° and 2θ of 40.5° denote amorphous regions, which are consistent with previous works by Peng et al. [24]. Figure 4b shows the PXRD pattern of ZIF-L nanosheets prepared in the absence of the cellulose matrix, which have been reported by Liu et al. [11]. These PXRD peaks, according to fitting patterns, agree with the spectrum of the prepared ZIF-L in this work and those ZIF-L in the reported works. The PXRD characteristic peaks of ZIF-L showed a semi-SOD architecture. It is apparent that ZIF-L has similar PXRD crystal features compared with ZIF-8 and a similar pore size of 3.4 Å inside the 2D MOF crystalline layers [9]. For CMC-ZIF 10 wt %, the value of 2*θ*, where ZIF-L PXRD patterns are clearly presented, are not overshadow by the wide peaks from the CMC. On the other side, by increasing the ZIF content, peak intensities of ZIF-L slowly improved for the PXRD spectrum of CMC–ZIF-L mixed membranes. Figure 4c,d indicates that the intensities of the PXRD peaks of CMC–ZIF-L mixed membranes at 2θ of 22.6° were weaker than that of the pure CMC membrane, revealing that the introduction of ZIF-L affected the chain packing of CMC as well as expanded the interchain spacing, therefore, reducing the crystalline degree. When the content was higher, the nucleation effect could be also limited by interfacial voids existing inside the mixed membranes, further decreasing the crystalline degree. 

**Figure 5:**
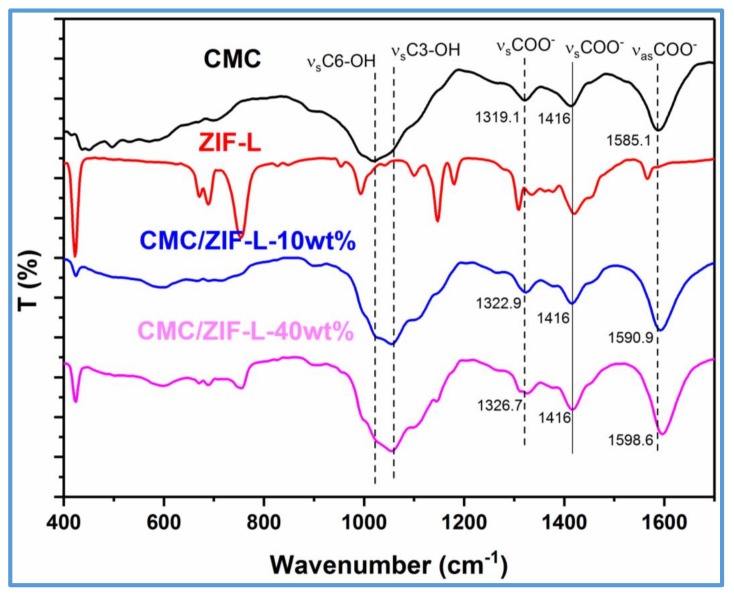
FTIR spectrum of the prepared membranes.

Figure 5 exhibits the FTIR curves of ZIF-L, CMC, and CMC–ZIF-L. For ZIF-L, the characteristic signals at 1584, 1146, around 749, and 422 cm^−1^ corresponded to the stretching vibration of C=N, the bending vibration of C-H, the bending vibration of the imidazole ring, and the vibration peak of Zn–N, respectively, which is in agreement with the reported work [11]. The characteristic peaks of ZIF-L presented the similar location and intensity as those of ZIF-8 due to the similar building blocks, which further reflected the similar chemical bonds [25]. Near a pH of 7.0, the carboxylic groups of CMC had been entirely deprotonated, an intense peak located at 1592 cm^−1^ related to carboxylate (COO^−^) asymmetric stretching, and two symmetric carboxylate stretches (1416 and 1324 cm^−1^) were obviously found [26]. The complex interactions in the CMC–ZIF-L composite were caused by the carboxylate ion to the bound ZIF-L. The downshift of the COO^−^ stretching peak from 1585 to 1598 cm^−1^ showed the COO^−^ groups binding to Zn ions. In the FTIR spectrum of CMC, a carboxylate asymmetric vibrational peak was detected, and a difference between the carboxylate groups in symmetric stretching was also detected with the appearance of two peaks around 1415 and 1324 cm^−1^. The interaction between the ZIF-L and the carboxylate groups of CMC generating a ZIF-L^+^-CMCCOO^−^ complex appeared, as measured by the signal shift distributed to the vibration of COO^−^ groups [27]. After addition of colloidal CMC solution to the flask, the CMC–ZIF-L suspension was immediately formed along with low soluble ZIF-L crystals. At the same time, the mixed suspension was stabilized in the solution by the CMC capping agent. However, because of the completed complex with ZIF-L in the aqueous suspension, the carboxylic groups of CMC interacted mainly with the Na^+^, causing a vibration peak nearer the original peak of CMC. The changed peak at 1023 cm^−1^ of the CMC/ZIF-L was found, which occurred owing to the C–O–C glycosidic ether links. This peak partially reduced due to ether bond cleavage when ZIF-L nanosheets were incorporated. Therefore, anionic CMC would interact with ZIF-L through both the negatively charged COO^−^ group and ionized OH group. 

**Figure 6:**
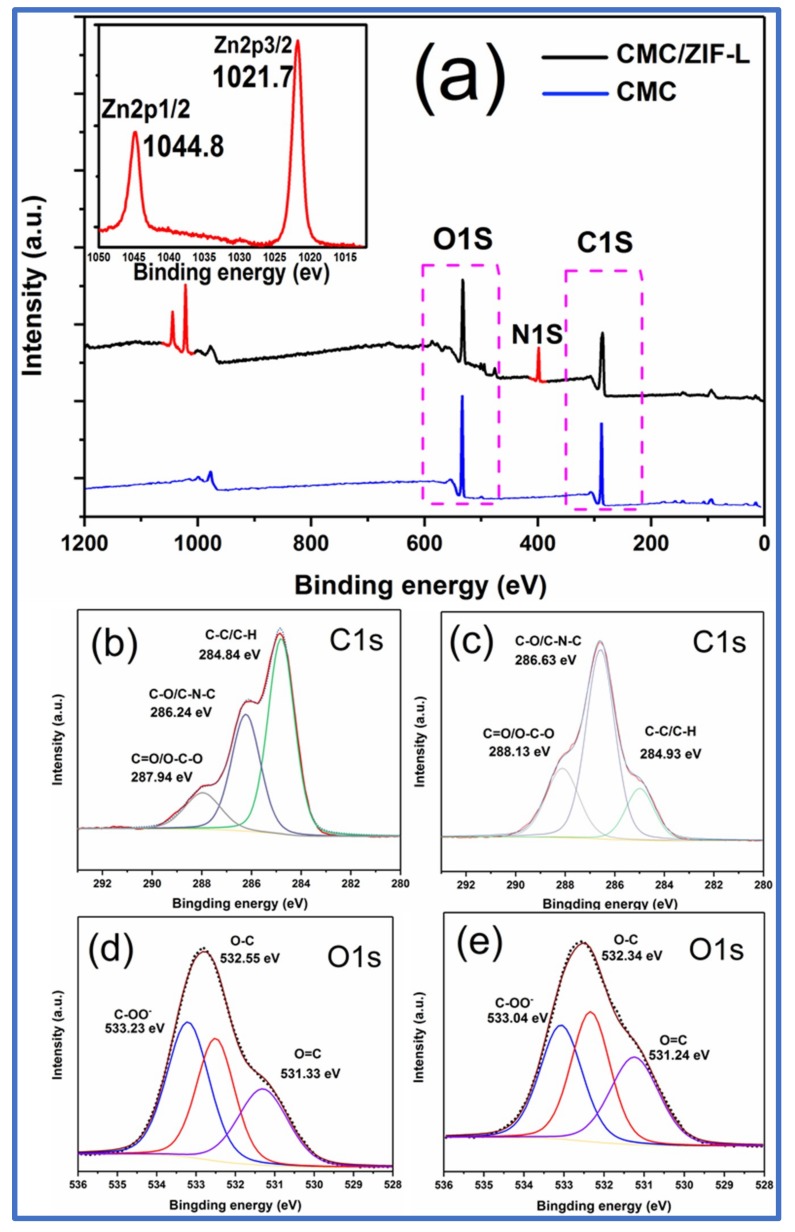
XPS spectra of (**a**) CMC and CMC–ZIF-L, (**b**) C 1s of CMC, (**c**) C 1s of CMC–ZIF-L, (**d**) O 1s of CMC, and (**e**) O 1s of CMC–ZIF-L.

To fully understand the functional groups and their interactions on the surface of the CMC–ZIF-L membrane, X-ray photo electron spectroscopy (XPS) measurements of CMC and CMC–ZIF-L membranes were carried out, as shown in Figure 6. Figure 6a shows the XPS spectra of CMC before and after ZIF-L incorporation. The wide scan spectra of Figure 6a show that CMC had two sharp peaks (C 1s, O 1s). It is quantitatively consistent with those reported by Matuana et al. [28] that these XPS spectra were in agreement with the chemical structure of the polysaccharide carboxymethyl cellulose (CMC) (Figure 6a), whereas CMC–ZIF-L had four peaks (C 1s, N 1s, Zn 2p, and O 1s). The existence of the Zn 2p peak at 1044.8 and 1021.7 eV demonstrated the successful incorporation of ZIF-L nanosheets into the CMC matrix. XPS spectra at the surface of the CMC–ZIF-L membranes in the C 1s, O 1s, and Zn 2p regions showed the existence of the organic shell of polysaccharide (CMC) used as a capping agent of ZIF-L nanosheets. As expected, for CMC, the spectra mainly indicated signals from carbon (C1s) and oxygen (O1s). Different chemical bonds of carbon atoms were shown by the C 1s spectrum: C–H and C–C bonds at a binding energy of 284.4 eV, a C–O bond at 286.3 eV, one peak at 287.8 eV assigned to C–O and O–C–O bonds, and O–C–OR bonds at 290.5 eV [29,30]. Peaks of O–C, O–C, and COO^−^ at 531.3, 532.3, and 533.1 eV, respectively, were observed in the O 1s region [27,31]. These were also examined by the changes in the binding energy of the bands associated with C–O, C–O, and COO^−^ in the spectra of C 1s (Figure 6c) and O 1s (Figure 6e) regions of CMC–ZIF-L compared with the pure CMC spectra. The O 1s spectrum signal included two peaks at 531.72 and 532.86 eV (Figure 6e), which can be assigned to zinc ions interacting with hydroxyl groups (Zn–OH). After ZIF-L incorporation, the area ratio of Zn–OH rose from 81.25% to 36.58%, showing the involvement of the hydroxyl group and the chemical interaction of CMC onto ZIF-L. The decreased peak at 533.4 eV also showed that Zn species interacted with carboxymethyl groups. 

**Figure 7:**
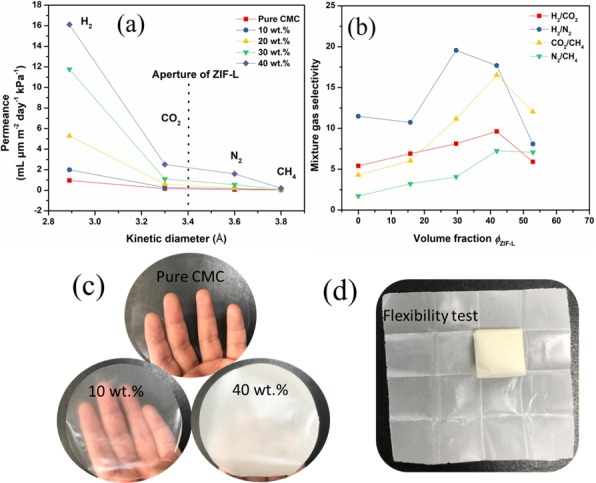
(**a**) Relation between kinetic diameters of the gases and their permeabilities through the CMC–ZIF-L membranes with different ZIF-L contents. (**b**) Relation between volume fraction of the composite membrane and their real mixture gas selectivities. (**c**,**d**) Optical image of CMC–ZIF-L membranes demonstrating their flexibility.

The CMC and CMC–ZIF-L membranes were tested by single H_2_, N_2_, CO_2_, and CH_4_ gases at 25 °C and the gas permeance performances are listed in Table 2. The differential pressure used in this study (100 kPa) was small enough, thus the tendency for CMC and CMC–ZIF-L membranes to swell and their aperture sizes to be distorted was too minor to be considered under such mild conditions. Figure 7a depicts the gas permeabilities plotted against the kinetic diameters of the permeate gases. As has been illustrated previously, nanoporous MOF nanosheets scattered in a polymer matrix present a certain degree of oriented stacking with greater exposure of crystal planes toward the gas concentration gradient, thus favoring selective gas permeation. The six-membered ring windows and four-membered windows of ZIF-L in the [200] plane are shown in Figure 1. These six-membered ring windows were of the same size as ZIF-8 and the value of the size was estimated by rigid framework models to be 3.4 Å, which is larger than the kinetic diameter of H_2_ (2.89 Å) and CO_2_ (3.3 Å), but smaller than N_2_ (3.64 Å). Because of the very small size, the effect of the four-membered ring windows on mass transport through the pore network was presumably negligible. Therefore, the ZIF-L was of a similar H_2_ permeance to ZIF-8 membranes and had a high selectivity of H_2_ over N_2_, CO_2_, and CH_4_. In previous studies, different kinds of matrix polymers for MOF composites were considered; however, undesirable gas diffusion occurred in the polymer matrix. Therefore, gas separation performance was limited. In contrast, for CMC, the high connection between the kinetic gas diameter and gas permeability showed that the behavior of gas permeation through the CMC layers could be explained with the help of the diffusion mechanism rather than the gas solubility in the layers. The highly dense structure of surface-charged, cellulose-based membranes had excellent gas barrier properties for most gases. A great number of hydrogen bonds between CMCs must contribute to constructing a highly dense structure. Furthermore, CMC membranes had high crystallinities and multiple hydrogen bonds, which provided ultradense structures (≈1.60 g cm^−3^). These CMC structures would probably greatly contribute to the good gas barrier properties; in this case, gases, including H_2_, N_2_, CO_2_, and CH_4_, could not easily pass through the pure CMC membrane. The influence of the loading amount was also investigated for CMC–ZIF-L membranes. Hybrid membranes containing 10, 20, 30, and 40 wt % loadings of ZIF-L nanosheets were prepared and their gas permeate performance was analyzed (Figure 7a). On the one hand, H_2_ permeability significantly increased at higher MOF loadings, reaching the highest value of 16.107 mL μm m^−2^ day^−1^ kPa^−1^ in CMC–ZIF-L-40 wt %, while only 0.965 mL μm m^−2^ day^−1^ kPa^−1^ could pass through the CMC membrane. This was expected, as higher MOF loadings caused a larger portion of nanopores in CMC–ZIF-L and free volume spaces, which facilitated H_2_ diffusion. Although N_2_ has a kinetic diameter bigger than the pore size of ZIF-L (3.64 vs. 3.4 Å), it was able to permeate across the CMC–ZIF-L membrane. This shows that the framework structure of ZIF-L is actually more flexible [10]. On the other hand, CH_4_ permeability was slightly improved from 0.036 to 0.199 when the ZIF-L nanosheets loading amount increased to 40 wt %.

The ideal separation factors are summarized in Table 3. By adding ZIF-L nanosheets, two different behaviors were found. First, for samples from 0–30 wt %, membrane permeation selectivity increased. The incorporation of MOF nanosheets of a 3.8-Å pore size indicated the molecular sieve property for the separation of these gases. Therefore, they inhibited the transmission of the larger molecule gases at a higher degree. However, it is noteworthy that the effect of H_2_/N_2_ separation for CMC–ZIF-L-10 wt % was significantly different from other samples. H_2_/N_2_ selectivity decreased from 14.48 to 12.83 compared with its H_2_/CO_2_ selectivity, while data from other samples all increased. This result could be explained by the synergistic effects of ZIF-L nanosheets embedded in the CMC matrix; that is, except for the gas leakage situation, gas permeation through the mixed membrane had no linear relationship with its ZIF-L loadings, especially for the sample with a relatively low ZIF-L content. 

In Figure 7a, by increasing the amount of ZIF-L inside CMC membranes from 0–30 wt %, the permeance of H_2_ and CO_2_ increased significantly, especially for H_2_. However, for N_2_ and CH_4,_ especially, CH_4_ permeation fluxes were limited, indicating that the ZIF-L addition had, respectively, increased both selectivity and permeability. These results show that the CMC–ZIF-L membrane could completely separate gases based on their molecular sizes, and no gas diffused into the CMC matrix. However, for mixed gas separation, H_2_/CO_2_ selectivity increased from 5.41 to 9.62, despite CO_2_ having a kinetic diameter of only 3.3 Å, which is smaller than ZIF-L (3.4 Å). This behavior was caused by the CO_2_ adsorption properties of the ZIF-L. The 2D layers of ZIF-L are joined by the terminal Hmim-4 and “free” Hmim-5. It is possible for these molecules to strongly coordinate with CO_2_ gas, and that results in an unusual decrease in the CO_2_ permeation rate. Contrary to the primary behavior, in the second behavior with the growth of nanosheets from 30 to 40 wt %, while the permeability of H_2_ was raised from 0.965 μm m^−2^ day^−1^ kPa^−1^ to 16.107 μm m^−2^ day^−1^ kPa^−1^, the selectivity for all gases decreased. In this situation, the particles aggregated together, which led to an inappropriate distribution inside the CMC matrix. As a result, membrane selectivity was greatly reduced. The specific mixture gas separation data are shown in Table S1. Gas mixtures of H_2_/CO_2_, H_2_/N_2_, CO_2_/CH_4_, N_2_/CH_4,_ and H_2_/CO_2_ (50/50, 75/25, and 25/75 vol %) were used to test CMC-ZIF-L composite membranes with 30 wt % ZIF-L nanosheets loadings, as can be seen in Table S2. In contrast, the same tendencies can be observed, and the variations in their selectivities are within the margin of error. This could be explained by the molecular sieving effect of ZIF-L crystals. Besides, this result suggests that there is no significant competitive permeation for the mixed gas pairs, also indicating the rigid framework structure of ZIF-L. Overall, it can be concluded that three factors make great contributions to the high gas selective permeation of CMC–ZIF-L membranes: (a) The high gas barrier properties of CMC membranes, (b) the ordered aperture diameter of ZIF-L nanosheets, and (c) the strong interactions between ZIF-L nanosheets and CMC.

## 4. Conclusions

In summary, we successfully fabricated a CMC crosslinked ZIF-L membrane by using CMC as the gas barrier and adhesive matrix through a facile, organic-solvent-free method. Combining carboxymethyl cellulose and ZIF-L nanosheets contributed to the high dispersion stability of ZIF-L nanosheets in an aqueous system due to the interactions between CMC and ZIF-L nanosheets as well as the anion charge repulsion effect between the complex particles. Based on the uniform CMC–ZIF-L suspension and good interactions between CMC and ZIF-L, the synthesized composite membrane with high ZIF-L nanosheet loadings exhibited ultraselective gas permeation properties. For CH_4_, the composite membrane showed an almost similar gas barrier behavior, with the pristine high gas barrier CMC membrane at 0% RH; for H_2_, the permeation of the CMC–ZIF-L membrane was 16.84 times of that of CMC membrane. A series of gas permeation tests also indicated that very little gas leakage occurred in the composite membrane; that is, gases could only pass through the porous structure of the ZIF-L nanosheets. Gas separation results demonstrated that CMC–ZIF-L with 30-wt % ZIF-L nanosheets content was the best and had the highest separation ability. All these showed that the synthesized materials are good candidates for gas separation applications. In addition, the simple synthesis technique of adopting gas barrier CMC as a capping agent and adhesive matrix for fabricating an MOF-based membrane could be extended to various systems.

## Figures and Tables

**Table 1:** Physical characteristics of CMC–ZIF-L composite membranes.

*R*_ZL_ (wt %) *^a^*	*ϕ*_ZL_ (vol %) *^b^*	*ρ*_t_ (g cm^−3^) *^c^*	*ρ*_b_ (g cm^−3^) *^d^*	*σ*^t^ (MPa) *^e^*
0	-	1.60	1.63	78.1
10	15.76	1.50	1.54	46.8
20	29.63	1.41	1.42	32.6
30	41.92	1.33	1.34	26.3
40	52.88	1.26	1.28	21.5
100	-	0.95	-	-

*^a^* Weight fraction of ZIF-L *^b^*; volume fraction of ZIF-L *^c^*; theoretical density of the mixed membranes calculated by the mass ratio of ZIF-L and CMC *^d^*; bulk density calculated by weight and volume values of the mixed membranes *^e^*; samples were kept at 50% RH for 24 h before the tensile strength measurement.

**Table 2:** Pure gas permeation properties of CMC–ZIF-L composite membranes with different amounts of ZIF-L nanosheet loadings.

*R*_ZL_ (wt %) ^a^	Permeance (mL μm m^−2^ day^−1^ kPa^−1^) ^b^			
	H_2_	CO_2_	N_2_	CH_4_
0	0.956	0.162	0.066	0.036
10	1.989	0.276	0.155	0.043
20	5.278	0.611	0.213	0.049
30	11.767	1.108	0.554	0.062
40	16.107	2.514	1.595	0.199

^a^ Weight fraction of ZIF-L in the composite membrane. ^b^ Gas permeate performance.

**Table 3:** Ideal gas selectivity of CMC–ZIF-L composite membranes with different amounts of ZIF-L nanosheet loadings.

R_ZL_ (wt %) ^a^	Ideal Selectivity ^b^			
	H_2_/CO_2_	H_2_/N_2_	CO_2_/CH_4_	N_2_/CH_4_
0	5.90	14.48	4.5	1.83
10	7.21	12.83	6.48	3.6
20	8.63	24.77	12.47	4.55
30	10.62	21.54	17.87	8.93
40	6.40	10.09	12.63	8.01

^a^ Weight fraction of ZIF-L in the composite membrane. ^b^ Ideal gas selectivity according to Table 2.

## Supplementary Materials

The following are available online at http://www.mdpi.com/2073-4360/10/12/1340/s1, Figure S1: TGA curves of CMC, ZIF-L, and the mixed membranes, Table S1: Mixture gas selectivity of CMC–ZIF-L composite membranes with different amount of ZIF-L nanosheet loadings, Table S2: Mixture gas selectivity of CMC-ZIF-L composite membranes with 30 wt % ZIF-L nanosheets loading at different gas volume ratios.
