# Supplementary material for: Mixed Membranes Comprising Carboxymethyl Cellulose (as Capping Agent and Gas Barrier Matrix) and Nanoporous ZIF-L Nanosheets for Gas Separation Applications
Source: Polymers (Basel). 2018 Dec 4;10(12):1340. doi: 10.3390/polym10121340 (PMC6401715; doi:10.3390/polym10121340)
Supplement: Supplementary file 1 [file polymers-10-01340-s001.pdf]

Supplementary Materials

Mixed Membranes Comprising Carboxymethyl Cellulose (as Capping agent and Gas Barrier matrix) and Nanoporous ZIF-L Nanosheets for Gas Separation Applications

Fang Zhang, Jing Dou and Hui Zhang *

Jiangsu Provincial Key Lab of Pulp and Paper Science and Technology, Nanjing Forestry University, Nanjing, Jiangsu Province, 210037, China

* Correspondence: zhnjfu@163.com

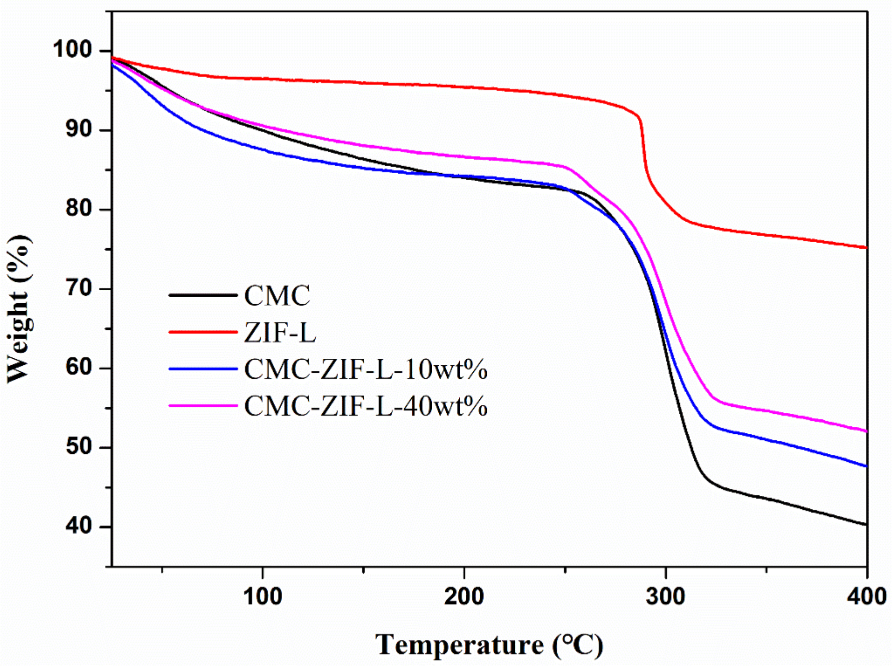

Figure S1. TGA curves of CMC, ZIF-L and the mixed membranes.

Table S1. Mixture gas selectivity of CMC-ZIF-L composite membranes at 50:50 vol.% with different amount of ZIF-L nanosheets loadings.

R_{ZL} (wt.%)[a]	Mixture gas selectivity			
	H_2/CO_2	H_2/N_2	CO_2/CH_4	N_2/CH_4
0	5.41	11.48	1.71	5.41
10	6.91	10.73	3.21	6.91
20	8.13	19.55	4.07	8.13
30	9.62	17.69	7.25	9.62
40	5.9	8.09	7.08	5.9

[a]Weight fraction of ZIF-L in the composite membrane.

Table S2. Mixture gas selectivity of CMC-ZIF-L composite membranes with 30 wt.% ZIF-L nanosheets loading at different gas volume ratios.

Vol. ratio [a]	Mixture gas selectivity			
	H_2/CO_2	H_2/N_2	CO_2/CH_4	N_2/CH_4
50/50	9.62	17.69	7.25	9.62
75/25	9.83	17.85	7.11	9.61
25/75	9.31	17.81	7.34	9.56

[a]Volume ratios of mixed gas in the feeding side.
